# The effect of climate change on the escape kinematics and performance of fishes: implications for future predator–prey interactions

**DOI:** 10.1093/conphys/coz078

**Published:** 2019-11-07

**Authors:** Paolo Domenici, Bridie J M Allan, Christel Lefrançois, Mark I McCormick

**Affiliations:** 1 CNR-IAS, Oristano, 09170 Italy; 2 Department of Marine Science, University of Otago, Dunedin 9054, New Zealand; 3 UMR 7266 LIENSs, University of La Rochelle—CNRS, La Rochelle 17000, France; 4 Department of Marine Biology and Aquaculture, ARC Centre of Excellence for Coral Reef Studies, James Cook University, Townsville, Queensland 4811, Australia

**Keywords:** Escape response, fish, global warming, hypoxia, ocean acidification, predator–prey interactions

## Abstract

Climate change can have a pronounced impact on the physiology and behaviour of fishes. Notably, many climate change stressors, such as global warming, hypoxia and ocean acidification (OA), have been shown to alter the kinematics of predator–prey interactions in fishes, with potential effects at ecological levels. Here, we review the main effects of each of these stressors on fish escape responses using an integrative approach that encompasses behavioural and kinematic variables. Elevated temperature was shown to affect many components of the escape response, including escape latencies, kinematics and maximum swimming performance, while the main effect of hypoxia was on escape responsiveness and directionality. OA had a negative effect on the escape response of juvenile fish by decreasing their directionality, responsiveness and locomotor performance, although some studies show no effect of acidification. The few studies that have explored the effects of multiple stressors show that temperature tends to have a stronger effect on escape performance than OA. Overall, the effects of climate change on escape responses may occur through decreased muscle performance and/or an interference with brain and sensory functions. In all of these cases, since the escape response is a behaviour directly related to survival, these effects are likely to be fundamental drivers of changes in marine communities. The overall future impact of these stressors is discussed by including their potential effects on predator attack behaviour, thereby allowing the development of potential future scenarios for predator–prey interactions.

## Teaser

**Lay summary**: Climate change in the aquatic environment is leading to global warming, OA and an increase in hypoxic areas and events. Here, we review the effects of these three major stressors of climate change on the escape response of fishes and their implications for predator–prey relationships.

## Introduction

A rapid increase in atmospheric carbon dioxide combined with anthropogenic pollutants is causing major changes in the physical and chemical properties of the ocean, with major impacts on its inhabitants. Climate change models project that ocean *p*CO_2_ will exceed 900 ppm by 2100 (Meinshausen *et al.*, 2011) from current levels of ~ 400 ppm, leading to a decrease in ocean pH. Elevation of this primary greenhouse gas will also lead to a predicted 3–5°C increase in ocean temperatures over the same time period. The temperature dependence of chemical reactions means that levels of available oxygen within the shallow ocean will decrease, particularly when combined with increased levels of nutrient discharge along the shores and eutrophication (Breitburg *et al.*, 2018), and these have already led to an increase in the size and number of hypoxic zones in coastal and oceanic waters. In the aquatic environment, global warming, ocean acidification (OA) and hypoxia are intrinsically linked with each other and with an array of other stressors (e.g. heavy metals, plastics, petrol chemicals) that are predicted to increase over time due to accelerating global economies (Halpern *et al.*, 2007). Global warming can increase the release of CO_2_ from terrestrial and marine sinks, thereby creating a positive feedback causing a further increase in temperatures (Doney *et al.*, 2009). Similarly, global warming is contributing to deoxygenation, as a result of a decrease in oxygen solubility and an increase in oxygen consumption in marine organisms (Breitburg *et al.*, 2018). OA is predicted to be amplified by hypoxia in coastal areas, where much higher *p*CO_2_ values than predicted for other areas of the ocean can be expected, because of the production of CO_2_ related to heterotrophic degradation of organic material (Melzner *et al.*, 2013). Furthermore, although the levels of each of these three fundamental stressors is known to show a natural daily and seasonal variation (Crabbe, 2008; Frölicher *et al.*, 2009; Hofmann *et al.*, 2011), the extent of their variation is predicted to increase, particularly for CO_2_ (Schulz and Riebesell, 2013; Shaw *et al.*, 2013; Pacella *et al.*, 2018) and temperature (Wang and Dillon, 2014).

Increased environmental fluctuations and climate change are posing major challenges to marine organisms. Climate change can affect their relative abundance and distribution as a result of their different physiological tolerances (Pörtner and Farrell, 2008). As a consequence, latitudinal shifts as well as mortality events of several marine species have been observed during the last few decades (Perry *et al.*, 2005; Coma *et al.*, 2009; Sunday *et al.*, 2012; Poloczanska *et al.*, 2013). However, recent work has suggested that the effect of climate change on marine organisms will be more complex than suggested by the relationship between each stressor and individual performance (Harley *et al.*, 2006). This is largely because in addition to affecting the physiology and performance of a given species, climate change is also likely to affect the relationship between species (Harley *et al.*, 2006; Gilman *et al.*, 2010), particularly the interactions between predators and their prey (Wilmers *et al.*, 2007; Nagelkerken and Munday, 2016; Draper and Weissburg, 2019). It is therefore of fundamental importance to study the effect of climate change on predator–prey interactions in order to increase our ability to predict how climate change will affect marine communities (Hunsicker *et al.*, 2011).

The outcome of predator–prey interactions is determined by their relative sensorimotor performance (Cooper and Blumstein, 2015). Although some research has been carried out on encounters between real predators and prey, in fishes, the escape response has typically been the focus of most studies on predator–prey interactions, largely because of the ease of the experimental approach (Domenici *et al.*, 2011). Fish escape responses typically consist of two muscular contractions followed by a glide or continuous swimming (Weihs, 1973; Domenici and Blake, 1997). The response is usually controlled by a pair of giant neurons located in the hindbrain of most fish species, the Mauthner cells, which receive multimodal sensory input and ensure short latency responses (Eaton *et al.*, 2001; Kohashi and Oda, 2008). Previous work has shown that the early stages of an escape response are fundamental in avoiding predation (Walker *et al.*, 2005; Catania, 2009; McCormick *et al.*, 2018a). In fishes, escape performance can be assessed by multiple traits that include both locomotor (e.g. speed, acceleration) and non-locomotor components, primarily related to behavioural and sensory performance such as the timing, the distance and the directionality of the response (Domenici, 2010a). Traits such as speed and acceleration of the escape responses are related to locomotor performance, hence muscle power and to some extent also neural control (Wakeling, 2006; Domenici, 2010b). On the other hand, predator and prey detection, reaction distances (i.e. the distance between the predator and prey at the time an escape is initiated, also called Flight Initiation Distance, FID, Cooper and Blumstein, 2015), the apparent looming threshold [ALT, i.e. the threshold looming rate of the approaching predator that triggers a response in the prey (Dill, 1974)], response latencies and the directionality of escape are related to neural and sensory capacity (Eaton *et al.*, 2001; Domenici, 2010a). The relative importance of each trait in determining the outcome of a given predator–prey interaction is likely to be context and species dependent (Domenici, 2010a; Domenici and Hale, 2019). For example, while speed was found to be the main determinant of escape success of guppies evading their natural cichlid predator (Walker *et al.*, 2005), in other predator–prey pairs, the main factor was found to be reaction distance (Scharf *et al.*, 2003; Nair *et al.*, 2017) or responsiveness (Fuiman *et al.*, 2006; McCormick *et al.*, 2018a).

Given the importance of predator–prey interactions in governing the distribution and abundance of marine fishes (Hunsicker *et al.*, 2011), it is fundamental to gain an understanding of how climate change can affect their escape and attack performance. This review focuses primarily on the effect of the ‘deadly trio’ of stressors (Bijma *et al.*, 2013), i.e. warming, acidification and hypoxia, on escape kinematics and performance, because prey have been the focus of most predator–prey studies carried out during the last couple of decades. Typically, prey responses have been studied using an artificial startling stimulus and their performance has been assessed under various levels of a given stressor. While this is a simplistic and reductionist approach, its advantage resides in assessing prey behaviour without the effect of the variability in the predator behaviour. The review will also discuss recent work based on more complex and realistic settings, in which a real predator attacking a prey was used, under various combinations of multiple stressors and, in some cases, using fluctuating levels mirroring those found in nature. Finally, we will discuss future avenues for research in which we advocate an increase in the complexity of the experiments, in terms of multiple stressors, environmental fluctuations, realistic predator–prey settings and trans-generational experiments, as well as the development of a modelling approach, in order to increase our ability to make predictions on how climate change will affect the ecology of predator–prey interactions.

## The effect of temperature

Global warming is having a major effect on the structure and functionality of aquatic communities, affecting the abundance and geographical distribution of aquatic organisms (Daufresne *et al.*, 2009; Sunday *et al.*, 2012; Poloczanska *et al.*, 2013). Ecological effects at the population, community and ecosystem level have been claimed to be largely related to the physiological responses of aquatic organisms to temperature (Helmuth *et al.*, 2005; Pörtner and Knust, 2007; Pörtner and Farrell, 2008; Chown *et al.*, 2010). Fishes, like all other ectotherms, are particularly vulnerable to temperature changes (Pörtner and Farrell, 2008; Isaak and Rieman, 2013) and therefore, it is important to understand how temperature affects their overall performance as well as their inter-specific interactions, in order to allow predictions of how climate change will affect aquatic communities.

Many organismal functions are temperature dependent, and thermal response curves have been derived that illustrate the relationship between various fundamental biological rates (such as growth, metabolic scope, metabolic rate, growth, activity and reproduction) and temperature (Wood *et al.*, 1997; Fenkes *et al.*, 2016). In addition to such basic physiological traits, swimming performance and kinematics are known to be affected by temperature due to alterations in aerobic activity and endurance (Claireaux *et al.*, 2000; McKenzie and Claireaux, 2010; Johansen and Jones, 2011; Lea *et al.*, 2016), cardiac output (Eliason *et al.*, 2011), muscle development (Hanel and Wieser, 1996) as well as power output for anaerobic swimming (burst) through changes in the contractile properties of the swimming muscles (Wakeling, 2006). Here, we discuss how temperature may specifically affect escape swimming performance and kinematics, which are relevant for predator–prey interactions. In addition, temperature can affect other traits that are key in predator–prey interactions, such as those related to brain and sensory functions, e.g. the timing of the response (Preuss and Faber, 2003). As a result, temperature is likely to change the balance of predator and prey performance, which can have a major effect on aquatic communities at large.

### Temperature effects on escape locomotion

Temperature can have major direct or indirect effects on swimming performance and consequently on predator–prey interactions. The indirect effects are related to the well-known relationship between temperature and the general condition of the fishes, i.e. growth rate (i.e. size), condition factor, muscle content, development and reproductive status (Pauly, 1980; Pankhurst *et al.*, 1996; Pörtner, 2002; Garcia de la serrana *et al.*, 2012; Ackerly and Ward, 2016). All of these factors can affect the overall performance of fishes including its swimming kinematics (James and Johnston, 1998a,b). Importantly, an increased size, due to temperature-induced faster growth rate, can cause a higher speed because of the positive relationship between size and burst speed (James and Johnston, 1998a; Domenici, 2001). Therefore, the effect of temperature on factors such as development, body size and condition can indirectly cause changes in burst swimming, such that warming may also indirectly increase burst swimming performance (Martínez *et al.*, 2004; Álvarez and Metcalfe, 2007; Ackerly and Ward, 2016; Watson *et al.*, 2018). On the other hand, the direct effects of temperature on escape swimming performance are mainly due to the effect of temperature on muscle performance and power output, which tend to increase at higher temperatures (Wakeling, 2006; Wilson *et al.*, 2010; James, 2013). Acute decreases in temperature can result in a decrease in body bend and muscle strain rate during escape responses, while an acute increase in temperature increases muscle shortening speed, causing greater power output and higher escape swimming performance (Wakeling *et al.*, 2000).

It is likely that alterations in performance will be most apparent when temperature changes occur over a short time period (i.e. acute), while acclimation can compensate for the effect of temperature on escape swimming to a large degree when temperatures change slowly, such as during seasonal temperature shifts in mean temperature (Beddow *et al.*, 1995; Johnson and Bennett, 1995; Temple and Johnston, 1998; Wilson *et al.*, 2010). The extent of this compensation depends on the duration of the acclimation, is largely species and habitat specific, and is related to thermal tolerance ranges, such that species with a wide thermal range (e.g. goldfish *Carassius auratus*) tend to show greater compensation in escape swimming (e.g. speed, distance travelled, turning rates) than species with a narrow thermal range (e.g. killifish *Fundulus heteroclitus*) after 4 weeks of acclimation (Johnson and Bennett, 1995). Similarly, in Trinidad guppies (*Poecilia reticulata*) and Mediterranean grey mullets (*Liza aurata)*, the escape speeds of fish acclimated (for 70 days and 1 month, respectively) to each temperature did not differ, as a result of complete compensation (Muñoz *et al.*, 2012; Killen *et al.*, 2015). In common carp (*Cyprinus carpio*), 2 weeks of acclimation can induce changes at the anaerobic muscle fibre level and extend the range of temperature for escape swimming but do not result in full compensation (Wakeling *et al.*, 2000). In golden perch (*Macquaria ambigua)* tested within a range of 10–25°C after 3 weeks of acclimation, escape swimming performance decreased significantly at temperatures below approximately 15°C but was relatively constant between 15 and 25°C (Lyon *et al.*, 2008). The strength of the acclimation response can also be associated with seasonal variation in temperature, especially during development (Wakeling *et al.*, 2000). In mosquitofish, (*Gambusia holbrooki*) escape speeds did not differ between spring and summer fish at their natural water temperatures of 15 and 25°C, respectively (Seebacher *et al.*, 2014). While temperature acclimation in adults and juveniles is reversible, temperature effects during the early developmental stages can have more long-term effects on physical traits (Johnston and Temple, 2002) and can be reflected in burst swimming performance (Batty *et al.*, 1993).

Furthermore, increasing temperature can directly affect escape response by reducing the viscosity of water. In order to partition the effect of temperature into those due to change in viscosity and those due to effect on fish physiology, experiments on escape responses have been carried out by changing the viscosity of the water. Work on various species has shown that the effect of viscosity may be relevant for the escape swimming of small larvae at lower temperatures, while for larger larvae and higher temperatures, the effect of temperature on fish physiology is the main factor driving changes in swimming performance (Herbing, 2002). The escape speeds in large juvenile goldfish (*C. auratus*) (77 mm in body length) were not affected by increases in viscosity at constant temperature, while escape speeds in smaller guppies *(Poecilia reticulata)* (22 mm in body length) were reduced (Johnson *et al.*, 1998). Viscosity was found to have an overall effect on the escape response kinematics of larval zebrafish (*Brachydanio rerio*) (3.3 mm in body length), by significantly decreasing displacement, maximum velocity and acceleration during stage 1 (i.e. the first muscle contraction) of the escape response; however, the viscosity values used were outside the range of viscosity variation caused by temperature changes in nature (Danos and Lauder, 2012).

### Temperature effects on non-locomotor variables of the escape response

Temperature affects various non-locomotor components of the escape response, such as responsiveness, escape latency, reaction distance and directionality from the threat. Temperature has been known to affect escape latency, mainly as a result of the changes in the neural conduction speed, with the highest conduction speed occurring at high temperatures. Fish acclimated for 2 weeks at different temperatures, i.e. 5–25°C (Webb, 1978) and 15–25°C (Penghan *et al.*, 2016), showed shorter escape latencies at higher temperature. Szabo and colleagues acclimated goldfish (*C. auratus*) for 4 weeks at 5, 15 or 25°C and found that the higher temperature yielded the shorter latencies, suggesting that these effects were due, at least in part, to higher conduction velocity in the Mauthner axons of warm-acclimated populations (Szabo *et al.*, 2008). Furthermore, acclimation to high temperature was found to increase responsiveness, likely because fish became hyper-excitable as a result of a change in the balance between excitatory and inhibitory synaptic transmission (Szabo *et al.*, 2008). Although higher temperature may increase both responsiveness and the latency performance of fish, other escape traits such as directionality (the proportion of escape responses away from the threat) may be impaired (Szabo *et al.*, 2008). Szabo *et al.* (2008) suggest that decreased directionality was due to the higher conduction velocity resulting from high temperature in warm-acclimated population, which may reduce the temporal separation of inputs to the two Mauthner cells, causing reduced right–left discrimination. In contrast, work based on acute cooling found that low temperatures increase responsiveness and decreased directionality (i.e. by increasing the proportion of responses towards the threat) in goldfish (*C. auratus*) (Preuss and Faber, 2003). These studies show that acute cooling lowered the behavioural threshold for spike initiation in the Mauthner cell, presumably because of changes in the intrinsic properties of the Mauthner cells (Preuss and Faber, 2003). Similarly, acute warming (34–39°C) was found to decrease the reaction distance in goldfish (*C. auratus*) to an approaching threat compared to the control level (15°C), likely as a result of the malfunctioning of the neurosensory system (Webb and Zhang, 1994).

Each species’ capacity to acclimate to temperature change is likely to be dependent on the relative position of an individual on its thermal reaction norm, on the evolutionary history and geographical range of the species, the latitudinal/geographic location and on the thermal history of the test population. For example, while a short-term (4 days) exposure to high temperature was found to decrease directionality away from the threat in two species of coral reef fishes (*Pomacentrus moluccensis* and *Pomacentrus amboinensis*), directionality returned to control levels after a longer term (90 days) acclimation only in *P. moluccensis* (Warren *et al.*, 2017). However, escape latency was not found to be affected by temperature within the range of temperatures used (29–31°C) (Warren *et al.*, 2017). Seasonal variation in temperature was also found to affect responsiveness and reaction distance, since performance in these traits decreases towards the end of the spawning season (September to November) over a period of temperature decline (Ojanguren and Fuiman, 2010). Thermal conditions during development (i.e. higher incubation temperature) are also likely to have a positive effect on burst swimming performance (i.e. burst speed) and therefore on vulnerability to predation (Seebacher and Grigaltchik, 2015).

### Temperature effects on predator–prey encounters

Given all the effects that temperature can have on escape locomotion and behaviour, temperature is expected to modulate the outcome of predator–prey interactions. Species-specific differences, the duration of temperature acclimation, as well as the differences in temperature between the experimental treatments may modulate these effects. Because predators and prey may respond differently to climate change, the consequent impact on their interactions may have profound effects on population dynamics (Dell *et al.*, 2014). Temperature changes may not only alter predator attack and prey escape performance, for example through an effect of temperature on the muscle or sensory performance of the prey as discussed above, but also prey capture kinematics (Wintzer and Motta, 2004; Devries and Wainwright, 2006; Higham *et al.*, 2015). They may also indirectly affect the outcome of predator–prey encounters because temperature has a well-known effect on metabolism of the predators, with metabolic scope increasing with temperature up to an optimum, beyond which a decline is observed (Pörtner and Farrell, 2008). Temperature changes can, therefore, potentially alter predator activity and hunger level, hence their motivation to forage.

Studies on the effect of temperature on predator–prey interactions have used different acclimation periods, species and experimental temperatures. Different acclimation periods are ecologically relevant because of the temporally hierarchical nature of temperature fluctuations in aquatic systems. However, this diversity in the experimental approach is likely to be the main cause of some differences in the overall results, with longer acclimation periods typically showing a higher degree of compensation in performance. A week exposure to high temperature was found to increase the capture success of the fish predator (the dottyback, *Pseudochromis fuscus*) on fish prey in coral reef species (*Pomacentrus wardi*) (Allan *et al.*, 2015). This was likely due to a combination of effects on predators and prey. In term of the prey performance, reaction distance to the approaching predator and escape swimming decreased at high temperature. Conversely, attack rate and attack speed of the predator increased at high temperature. Allan *et al.* (2015) suggest that the effect on predator performance was likely due to an effect of temperature on motivation, since high temperature increases metabolic rate and thus the hunger level.

Other studies also suggest that temperature can affect not only locomotor performance but also the predator’s motivation to attack prey. Using 1 month of acclimation, Grigaltchik *et al.* (2012) showed that warm acclimated bass (*Macquaria novemaculeata*) had higher attack rates on mosquitofish (*Gambusia holbrooki*) than cold acclimated ones, and higher prey capture success. Interestingly, laboratory trials showed that the burst swimming performances of acclimated seabass did not differ between warm and cold temperatures, indicating that muscle function was maintained through acclimation, thus low capture rates at low temperature were likely to be due to decreased motivation to catch prey as a result of lower food requirement (Grigaltchik *et al.*, 2012). Furthermore, when tested at a temperature higher than the acclimation range, attack rates declined, while the escape swimming performance of the prey was higher than the attack speed of the predators. Therefore, in some predator–prey pairs, predator pressure may increase with temperature up to a point, and then it may decline again (Grigaltchik *et al.*, 2012), although this pattern will be modulated by acclimation time. A similar pattern was found in the relationship between temperature and strike rate of common coral trout (*Plectropomus leopardus*), from the Great Barrier Reef (Scott *et al.*, 2017). Hence, differences in the thermal reaction norms of performance and physiology between prey and predator will affect the outcome of interactions in response to temperature change.

**Figure 1:**
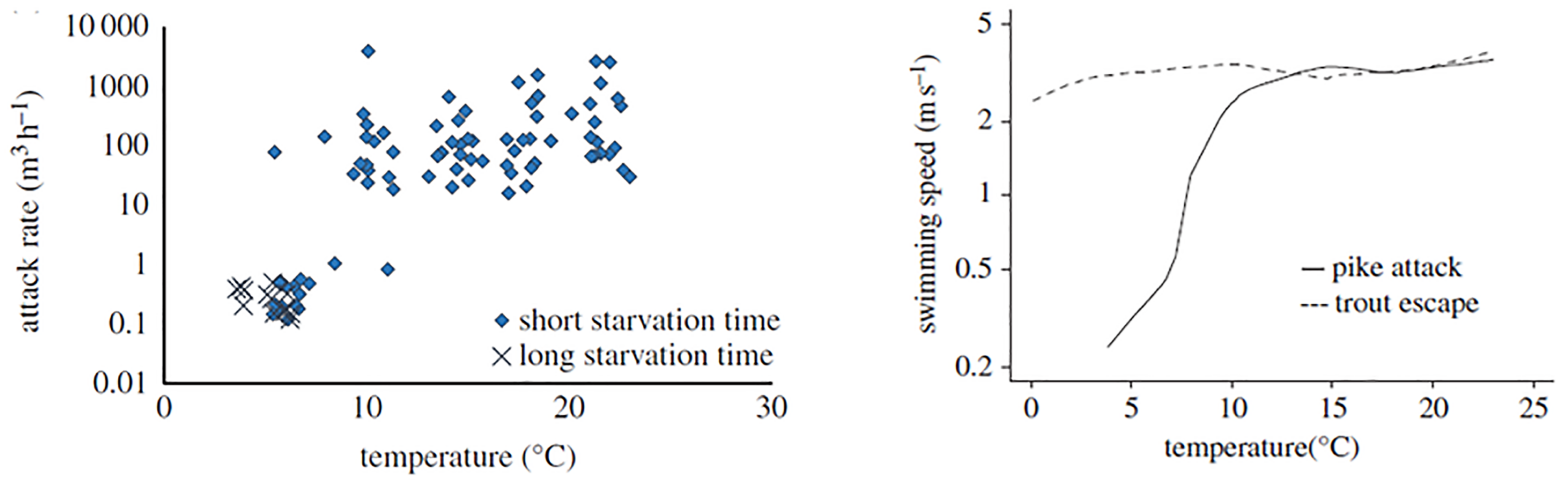
The differential effect of temperature on predators and prey. (**A**) The effect of temperature on the attack rate of northern pike (*Esox lucius*) attacking brown trout (*Salmo trutta*). Attack rates are expressed as m^3^ h^−1^, since they are measured as 1/*Pt*, where *P* is predator density and *t* is time to capture. (**B**) The effect of temperature on pike attacking speed and brown trout escape speed. From Öhlund *et al.* (2015).

**Figure 2:**
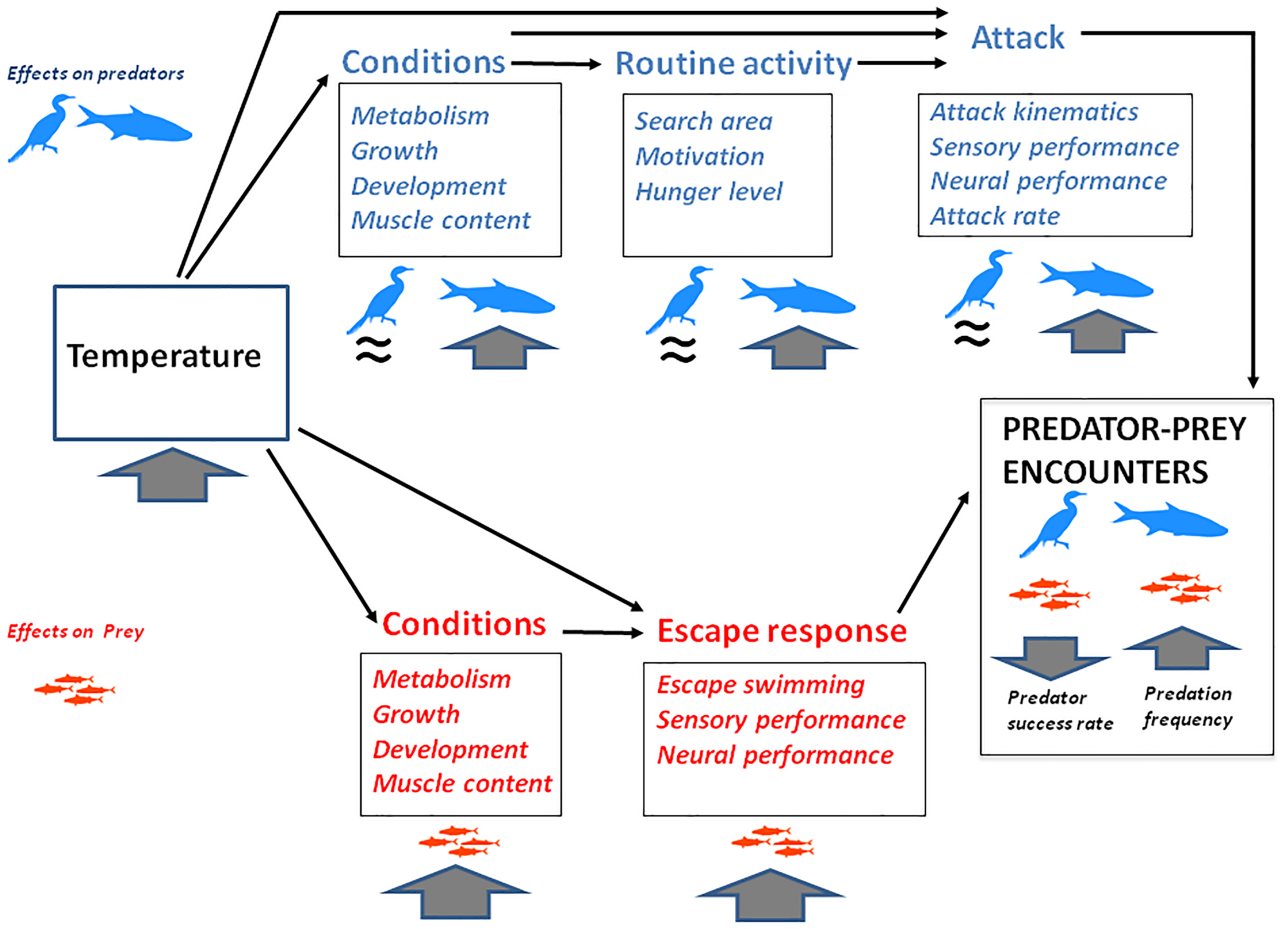
The potential effects of changes in temperature on predator–prey interactions, based on fish as prey, and fish or an endotherm (e.g. a bird) as predators, based on a scenario of future global warming. Downwards and upwards arrows indicate a decrease and an increase, respectively; while a ≈ sign indicates no significant change. At high temperatures, prey conditions (growth and metabolism) increase and so does the escape performance, also because of the direct effect of temperature. Temperature has similar positive effects on fish predator attacks (via direct effects and because of decreased body conditions). In addition, because of their higher routine activity and higher hunger level (due to higher metabolism), the frequency of predator–prey interactions increases (though they may decline at high temperatures that exceed acclimation temperatures; see main text). Temperature has little effect on performance and hunger level on endotherms (compared to the effect on ectotherms), and therefore predator success rate is expected to decrease at high temperature because of the positive effects of temperature on prey escape performance.

Further research has suggested a decrease in predator pressure at low temperatures. This is in line with Dell *et al.* (2014), who suggest that in ectotherm pairs of predators and prey at low temperatures, the escape speed of prey tends to remain close to peak levels and thus higher than the attack speeds of the predator. Work on pike (*Esox lucius*)—brown trout (*Salmo trutta*) interactions suggests that changes in relative speed performance were at the basis of the effect of long-term temperature exposure (3 months) on the outcome of the interactions (Öhlund *et al.*, 2015). Attack rates decreased sharply at low temperatures (below 11°C) and mirrored a decline in the attack speed of the predator, while escape speeds remained relatively constant (Fig. 1). Two potential explanations were put forward: (i) low temperature affects the neural performance of fish, which is likely to have a stronger effect on the predator than on the prey, because catching a fast-moving prey is a complex task that requires synchronization of motor and cognitive processes, while escaping mainly requires rapid random motion and (ii) low temperature reduces motivation, e.g. because of lower hunger and prey availability (Öhlund *et al.*, 2015). Overall, these studies suggest that at lower temperatures, predator–prey interactions involving fish as prey and predators tend to decrease in frequency (Fig. 2). In the case of endotherm predators (e.g. birds and marine mammals) attacking fish, the effects of temperature are more complex and do not usually result in a reduction of hunger and food intake in the same way as for ectotherms. For example, birds (cormorants *Phalacrocorax carbo carbo*) feeding in the winter economize energy expenditure by halving the time spent at sea, and halving the number but doubling the mass of each prey fish taken (Johansen *et al.*, 2001). Bailleul and colleagues suggest that after migrating to the Antarctic continent, elephant seal (*Mirounga leonina)* may target cold waters to facilitate the capture of fish prey that are less active at low temperatures (Bailleul *et al.*, 2007). It is therefore plausible that low-temperature conditions may increase the predation success rate of piscivorous endotherms (Fig. 2).

Accordingly, marine endotherms were suggested to be competitively favoured over ectothermic predators as water temperatures decline and this may be the basis for why predators such as marine mammals and birds show high phylogenetic diversity in cold, temperate latitudes while their diversity is low in warmer regions (Cairns *et al.*, 2008; Grady *et al.*, 2019). This pattern may be related to the asymmetric response in a number of traits (e.g. metabolic, sensory and locomotory rates), which is constant in endotherms while decreases with temperature in ectotherms (Cairns *et al.*, 2008; Dell *et al.*, 2014; Grady *et al.*, 2019). As a result, colder water is favourable to endothermic predators attacking slower ectothermic prey (Cairns *et al.*, 2008; Grady *et al.*, 2019). Arguably, the overall effects of global warming are bound to be more complex and beyond the scope of the current review, since they can have a number of other direct and indirect effects on aquatic organisms that would impact on predator–prey interactions, such as changes in the distributional ranges of both predators and preys (Learmonth *et al.*, 2006).

Overall, these studies suggest that temperature is a major factor determining the outcome of predator–prey interactions with fish as prey, although the type of change, and therefore, the potential future scenario will depend largely on whether the predators involved are endotherm or ectotherm (Fig. 2). Acclimation may in part compensate for the effect of temperature on some aspects of performance, although motivational components on the predator’s part appear to be a key element of the interactions. In addition, such complex interactions, in which motivational, sensory and locomotor factors can play a role, are unlikely to be driven by a single temperature effect. Regardless of the functional explanation for the effect on predator–prey interactions and the extent to which it is due to changes in escape or attack swimming, sensory performance, differences in predator hunger levels or a combination of all of these factors, studies of predator–prey interactions can be fundamental for predicting tipping points in the responses of ecosystems to global warming (Öhlund *et al.*, 2015).

In addition, while previous work shows that climate change stressors can have a number of effects on escape performance and therefore on prey vulnerability, further important effects of climate change (e.g. warming) may impact other aspects of swimming (e.g. aerobic swimming), which can trade off with anaerobic swimming (Reidy *et al.*, 2000), (but see Marras *et al.*, 2013), with potential consequences of sustained activity such as that used for migration. Therefore, the overall ecological effects of climate change on organisms need to take into account many interacting levels of physiological impacts that may result in a number of trade-offs.

## The effect of hypoxia

The increasing frequency of hypoxia events during the last decades makes hypoxia one of the most threatening stressors for aquatic organisms (Diaz, 2001; Breitburg *et al.*, 2018). While anoxia can be lethal for most aquatic organisms, hypoxia is known to induce many non-lethal effects. For example, hypoxia has a negative impact on fish growth (Chabot and Dutil, 1999), development (Shang and Wu, 2004), activity levels (Schurmann and Steffensen, 1994), muscle composition (Rees *et al.*, 2012), aerobic scope (Claireaux and Chabot, 2016) and aerobic swimming performance (Vagner *et al.*, 2008). In addition, as a result of hypoxia exposure, various short- and long-term adaptations can occur in fish, such as modifications in body and gill morphology (Sollid *et al.*, 2003; Crispo and Chapman, 2011). Since body morphology can affect escape locomotion (Domenici *et al.*,, 2008; Langerhans and Reznick, 2010), such modifications may potentially cause an indirect effect of hypoxia on predator–prey interactions.

Fish escape locomotion relies on fast anaerobic muscle fibres; therefore, hypoxia was originally hypothesized to have no effect on a single burst swimming event (Beamish, 1978). However, hypoxia is known to reduce the sensory performance in various vertebrate taxa, such as amphibians (Sitko and Honrubia, 1986), fishes (Robinson *et al.*, 2013), mammals, and other vertebrates (Fowler and Prlic, 1995). As a consequence, any effect of hypoxia on the sensory performance and neural control of escape response may also be reflected in diminished escape performance. In addition, hypoxia may limit the ability of fish to recover from anaerobic exercise, thereby limiting their potential to engage in repetitive bursts of activity. Here, we discuss how hypoxia may affect escape responses by considering both locomotor (e.g. speed and displacement) and non-locomotor traits (e.g. responsiveness and escape latency), as well as potential strategies employed by fish to minimize the negative effect of hypoxia, such as aquatic surface respiration, and the related behavioural trade-offs.

### The effect of hypoxia on escape locomotion

**Figure 3:**
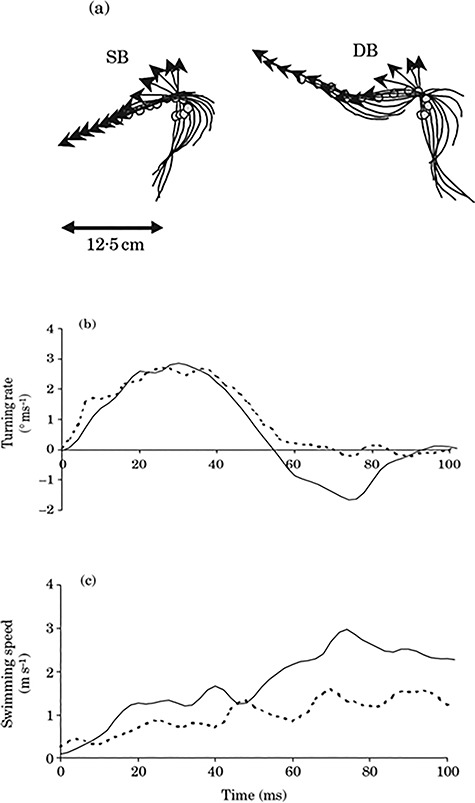
The escape response of golden grey mullets (*Liza aurata*). (**a**) Tracing of the midline and centre of mass (open circle) of golden grey mullets, shown at 10 ms of intervals. Top left, a single bend (SB) response in which fish glides after the first body bend (stage 1). Top right, a double bend (DB) response, in which fish performs two consecutive body bends. (**b**) the turning rate vs. time curve shows that DB responses (continuous line) reverse the turn (i.e. negative values), while the turning rate of SB response (dotted line) is around zero after stage 1 (i.e. after around 60 ms). (**c**) The curve of escape speed vs. time shows that speed is higher in DB (continuous line) than in SB responses (dotted line). DB responses are more common in normoxia than in hypoxia, in golden grey mullet. From Lefrancois *et al.* (2005).

In escaping golden grey mullet (*Liza aurata*), cumulative distance and maximum swimming speeds were found to be reduced following a few hours of exposure to acute hypoxia (Lefrançois *et al.*, 2005). This decreased escape performance was observed in severe hypoxia (i.e. 10% of air saturation) only when access to the surface was impeded experimentally. On the other hand, when *L. aurata* had access to the surface, they performed aquatic surface respiration (ASR, which allows fish to breath at the oxygenated layer near the air–water interface) and their performance did not differ from that of fish in normoxia (Lefrançois *et al.*, 2005). From a kinematic perspective, decreased escape swimming performance in this species was related to a decreased proportion of double bend responses, i.e. escape responses consisting of stages 1 and 2 (i.e. the first two body bends in an escape response, Domenici and Blake, 1997), as opposed to single bend responses in which only one body bend was observed and therefore lower speed (Fig. 3). Work on other species, however, did not find a significant effect of similarly acute hypoxia exposure on escape locomotion in European seabass (*Dicentrarchus labrax*) (Lefrancois and Domenici, 2006) or crucian carp (*C. carassius*) (Penghan *et al.*, 2014).

Zhang *et al.* (2013) and Ackerly *et al.* (2017) suggest that the duration of the exposure to hypoxia (i.e. chronic versus acute stress) may have a role in determining the behavioural response of fishes, although this is species specific. For example, grass carp (*Ctenopharyngodon idellus*) showed a decrease in maximum escape velocity only in normoxia-acclimated fish exposed to hypoxia, while no change was observed when fish were reared in similar hypoxic conditions (Zhang *et al.*, 2013). Ackerly and colleagues acclimated the African mormyrid (*Marcusenius victoria*) for 8 weeks to high and low dissolved oxygen (i.e. 20% of air saturation) before their escape swimming was tested under both these normoxic and hypoxic conditions. The results showed that the hypoxia-acclimated fish had shorter escape displacement when tested under hypoxia than in normoxia, while displacement of normoxia-acclimated fish was not affected by the test condition (Ackerly *et al.*, 2017). Gotanda and colleagues investigated the effect of long-term exposure to hypoxia in the African cichlid (*Pseudocrenilabrus multicolor victoriae*) (Gotanda *et al.*, 2012). Fish reared during at least 10 months in 15% air saturation-hypoxia developed larger gills, deeper bodies, and larger, wider heads than fish reared in normoxia. While different body morphologies could potentially have an effect on swimming performance, hypoxia-reared fish showed no difference in maximum velocity or acceleration compared to normoxia-reared fish. Gotanda *et al.* (2012) suggest that hypoxia-reared fish might compensate for morphological differences by using a high proportion of double-bend responses, to achieve similar performance as normoxia-reared fish. Overall, these results show that the effect of hypoxia on fish escape locomotion (i) is species specific, with some species showing decreased performance in hypoxia while other species show no effect; (ii) depends on acclimation and rearing conditions, with acclimation potentially allowing fish to partially compensate for exposure to hypoxic conditions in some cases; and (iii) may be affected by access to the water surface.

### Hypoxia effects on non-locomotor variables of the escape response

In fish escape responses, non-locomotor variables such as responsiveness, timing and response distances are known to affect vulnerability to predation (Domenici, 2010a). This is particularly relevant for the effect of hypoxia, because of the decreased brain and sensory performance in hypoxia observed in fish and other vertebrates (Fay and Ream, 1992; Fowler and Prlic, 1995; Robinson *et al.*, 2013). Hypoxia was found to reduce the responsiveness to a startling stimulus in both *D. labrax* and *L. aurata* exposed to an acute decrease of oxygen whether this latter species had access to the surface or not (Lefrançois *et al.*, 2005; Lefrancois and Domenici, 2006). A similar pattern was observed in the common sole (*Solea solea*) facing a progressive decrease in oxygen down to 15% air saturation over *c.* 1.5 h (Cannas *et al.*, 2012), as well as in grass carp (*Ctenopharyngodon idellus*) (Zhang *et al.*, 2013) and white grunts (*Haemulon plumieri*), even if hypoxic conditions were less severe (Sánchez-García *et al.*, 2017). Because responsiveness is a fundamental component of escape performance (Fuiman *et al.*, 2006), a decrease in responsiveness such as that observed in hypoxia may increase the risk of mortality and is expected to influence the outcome of predator–prey interactions.

Functionally, the hypoxia-related reduction in responsiveness may be due to an increase in response threshold, and/or decreased motivation to escape related to stress and exhaustion (Lefrançois *et al.*, 2005). Although most species investigated showed a decrease in responsiveness when exposed to hypoxia, the African mormyrid (*Marcusenius victoria*) showed no effect when exposed to acute or chronic hypoxia, suggesting that the species-specific differences in hypoxia tolerance are an important factor (Ackerly *et al.*, 2017). Species-specific difference were also found in the latency of the escape response; escape latency was not affected by hypoxia in *L. aurata* and *D. labrax*, while it increased in *C. idellus* (Zhang *et al.*, 2013) and decreased in *P.m. victoriae* (Gotanda *et al.*, 2012).

Directionality (i.e. whether a response is directed away or towards the threat, Domenici, 2010a) was found to be impacted by hypoxia in the two species in which directionality was tested [*L. aurata* (Lefrançois *et al.*, 2005) and *D. labrax* (Lefrancois and Domenici, 2006)]. Here, fish were exposed for a few hours to oxygen conditions lower than 20 and 50% of air saturation, respectively. In both cases, hypoxia-exposed fish showed a higher proportion of responses towards the threat. This is likely to result from an inaccurate left–right discrimination of the startle direction, possibly related to sensory disorientation (Lefrançois *et al.*, 2005). Because the early stages of an escape response are crucial for survival (Walker *et al.*, 2005; McCormick *et al.*, 2018a), an early mistake directing the prey towards the danger may increase its vulnerability.

### Effects of hypoxia on fish behaviour and predator–prey interactions

Aquatic surface respiration (ASR) and air breathing (AB) are performed by some fish species in order to avoid hypoxic conditions in the water column (Domenici *et al.*, 2007b). While ASR can reduce the negative effects of hypoxia on escape response as discussed above, it may also cause additional costs and risks (Domenici *et al.*, 2007b). Fish performing ASR and AB are at higher risk of predation owing to increased visibility to aerial predators (Kramer, 1987; Domenici *et al.*, 2007b), and the presence of predators can delay the onset of ASR (Shingles *et al.*, 2005). However, when performing ASR and AB, fish can decrease the risk of predation by surfacing with irregular frequency or in synchrony with others (Kramer and Graham, 1976). Irregularity reduces the predictability of the ASR and AB event, and therefore, the vulnerability to predation and synchronous surfacing was suggested to create sensory confusion in the predator (Kramer and Graham, 1976; Gee, 1980; Chapman and Chapman, 1994). Hypoxia is also known to increase the risk of predation by altering efficiency of anti-predator behaviours, such as school cohesiveness in pelagic species (Domenici *et al.*, 2002; Domenici *et al.*, 2007b) and crypsis in benthic species via changes in conspicuousness due to hypoxia-dependent increase in ventilation rates (Cannas *et al.*, 2012).

**Figure 4:**
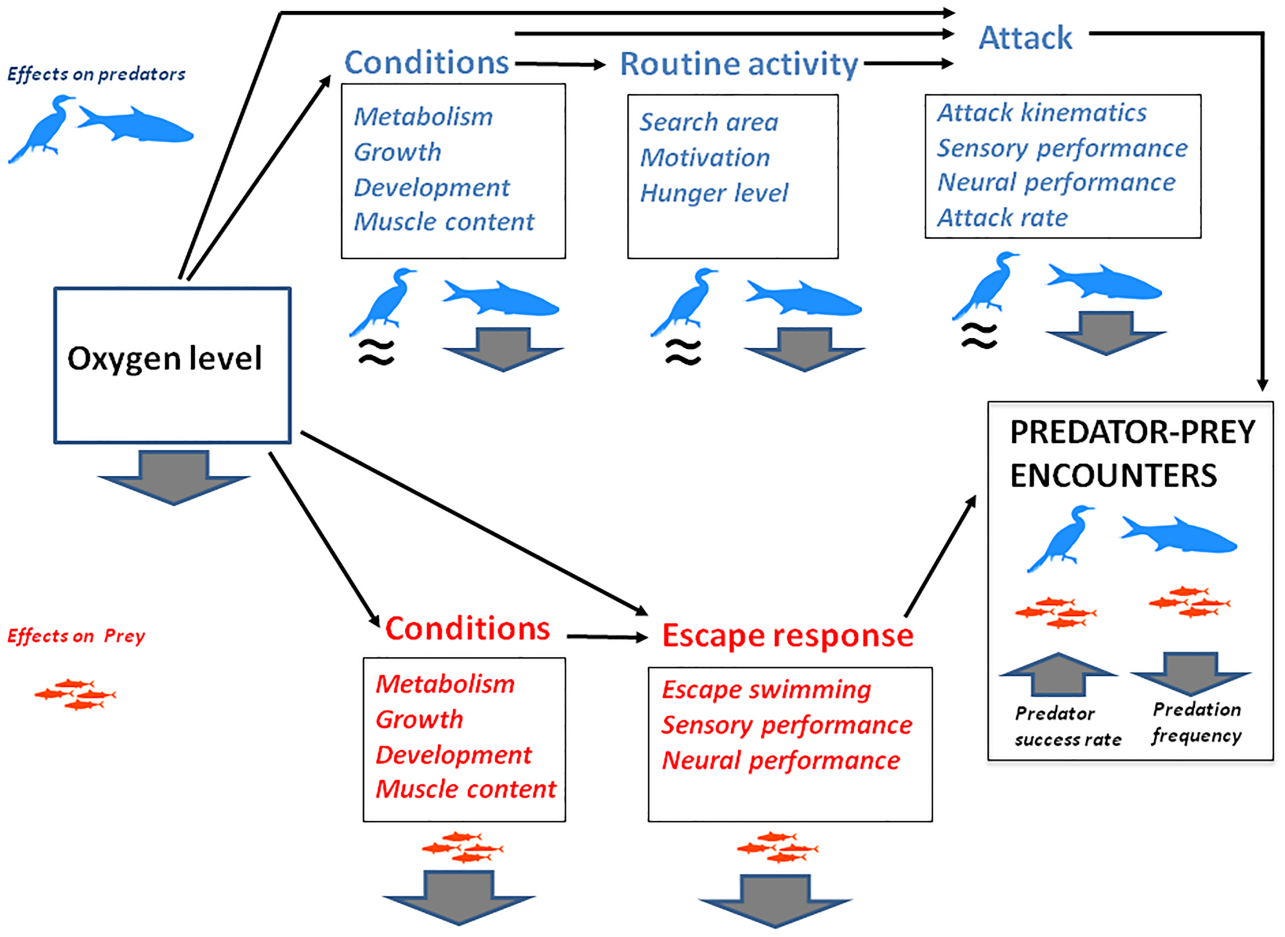
The potential effects of changes in oxygen level on predator–prey interactions, based on fish as prey, and fish or an endotherm (air breathing) as predators (e.g. bird), based on a scenario of future hypoxia. Downwards and upwards arrow indicate a decrease and an increase, respectively; while a ≈ sign indicates no significant change. When oxygen decreases, fish prey decreases their metabolism and growth, and their general conditions. This can have a negative impact on their locomotor and sensory performance. In addition to this indirect effect, hypoxia can also decrease escape performance directly (particularly non-locomotor performance). Hypoxia can also affect the conditions (growth and metabolism) of fish predator, but have no effect on endotherm, air-breathing predators such as birds. Similarly to fish prey, decreased conditions can impact the attack performance of fish predators, in addition to the direct (potential) effect of hypoxia on their performance in general. In addition, hypoxia-dependant decrease in metabolism decreases hunger level (thus motivation to feed and search activity) in fish predators, but not in birds. A possible scenario in fish–fish interactions is that the performance of both predators and prey decreases in hypoxia, and so does hunger level in predators. This could result in a decreased frequency of predator–prey events. On the other hand, birds could take advantage of the lower performance of fish prey in hypoxia, thus increasing their success rate in capturing a prey.

Similar to temperature, the effect of hypoxia on predator–prey interactions are taxon specific. Arguably, the effect of hypoxia on fish–fish interactions are likely to differ from those on predator–prey interactions involving fish as prey, and predators that are not affected by hypoxia, such as birds, aquatic mammals, or other hypoxia-tolerant taxa (Fig. 4). If the predators are fish, they are likely to be less hungry and feed less frequently than in normoxia (Poulin *et al.*, 1987; Chabot and Dutil, 1999; Thetmeyer *et al.*, 1999; Engström-Öst and Isaksson, 2006; Ripley and Foran, 2007), because of the reduced metabolism (Claireaux and Chabot, 2016) and possibly also decreased sensory performance (Robinson *et al.*, 2013). On the other hand, water hypoxia will have no effect on birds or marine mammals. As a result, predator pressure is likely to decrease in hypoxia when the predators are fish, while it should not change if the predators are unaffected by hypoxia. While there is both species-specific and methodology-specific (acute versus chronic) differences in the results of the effect of hypoxia on escape performance, most studies show some negative behavioural effects, particularly on directionality and responsiveness, while escape locomotion is affected only in some species (e.g. grey mullet; Lefrancois *et al.*, 2005). As a result, escape performance may decrease largely because of a decreased non-locomotor performance. Therefore, a potential scenario for fish–fish interactions is that the frequency of predation events should decrease in hypoxia (Fig. 4), with the exception of fish predators that are highly hypoxia tolerant such as certain air-breathing species (Wolf and Kramer, 1987). This is in line with work based on fish–fish interactions, which shows that the number of fish prey larvae consumed by Spanish mackerel (*Scomberomorus niphonius*) (Shoji *et al.*, 2005), as well as by juvenile striped bass (*Morone saxatilis*) and adult naked goby (*Gobiosoma bosc*) (Breitburg *et al.*, 1994) decreased in hypoxia. Conversely, predation by species that are hypoxia independent or highly hypoxia tolerant is expected to increase in low oxygen conditions, because prey escape performance in fish tends to decrease, while hunger in these predators will not be affected (Fig. 4). Indeed, works using hypoxia-tolerant predator species such as sea nettle (*Chrysaora quinquecirrha*) (Breitburg *et al.*, 1994) or moon jellyfish (*Aurelia aurita*) (Shoji *et al.*, 2005) show that predation on fish increases in hypoxia. Furthermore, aquatic birds such as little egrets (*Egretta garzetta*) were observed to take advantage of the ASR behaviour some lagoon fish undertake in the morning, when their surfacing in hypoxic waters (i.e. due to daily fluctuation in oxygen levels) makes them easy prey for birds (Kersten *et al.*, 1991).

While these potential scenarios are generally supported by previous work on predator–prey interactions in hypoxia, it is worth noting that hypoxia can also be associated to some behavioural strategies by the predators or the prey to circumvent the constraints imposed by hypoxia. For example, while predation by fish tends to decrease in hypoxia, certain fish species living in stratified waters routinely undertake brief foraging forays into hypoxic habitats to catch their invertebrate prey, suggesting that foraging in hypoxic waters may be common in fish when prey abundance is low in surface waters (Rahel and Nutzman, 1994; Taylor *et al.*, 2007; Roberts *et al.*, 2012). In addition, even within fish species, there can be differences in hypoxia tolerance (Bell and Eggleston, 2005), and it has been suggested that small fish tend to be more hypoxia tolerant that large ones, which may imply that fish prey tend to be more hypoxia tolerant than their fish predators (Robb and Abrahams, 2002; Robb and Abrahams, 2003; Pan *et al.*, 2016). Indeed, certain species of small fish were suggested to use deep hypoxic waters as a refuge from large predators (Chapman *et al.*, 1996a,b; Parker-Stetter and Horne, 2009; Ekau *et al.*, 2010; Hedges and Abrahams, 2015; Vejřík *et al.*, 2016). Clearly, hypoxia affects both the feeding and escape behaviour of predators and prey, as well as their habitat selection. Therefore, the complexity of the system is largely dictated by the relative hypoxia tolerances of each species involved and a modelling approach could be useful to improve our predictive ability of the effect of hypoxia on predator–prey interactions (de Mutsert *et al.*, 2016). Future studies may benefit from emphasizing such aspects, and incorporating the different capacities that prey and predators may have for acclimating and/or adapting to the more frequent and severe hypoxia episodes that are predicted.

## The effect of acidification

Climate change models project that ocean pCO_2_ will exceed 900 ppm by 2100 (Meinshausen *et al.*, 2011) from current levels of ~ 400 ppm, leading to a decrease in ocean pH, which occurs via the dissolution of CO_2_ as it is absorbed into the world’s oceans, where it is hydrated to form carbonic acid. Elevation of this primary greenhouse gas will also lead to a predicted 3–5°C increase in ocean temperatures over the same time period. The temperature dependence of chemical reactions means that levels of available oxygen within the shallow ocean will decrease. OA is predicted to affect interspecific interactions through changes in behaviour, locomotor and sensory performance (Wittmann and Pörtner, 2013; Nagelkerken and Munday, 2016). While early work on the effects of OA was mainly focused on calcifying organisms (Orr *et al.*, 2005), research in the last decade has shown that a number of behavioural disruptions in fish and marine invertebrates can occur as a consequence of exposure to high CO_2_ levels (Nagelkerken and Munday, 2016). Recent meta-analytical work has found various effects of exposure of high CO_2_ effects on calcification, metabolic rates and behavioural performances in fishes, in addition to increased predation risk and decreased foraging, particularly for fish larvae (Cattano *et al.*, 2018). With respect to predator–prey interactions, one of the most relevant effects is the disruption of the ability to avoid predator odours, as it was found in coral reef fish larvae (Dixson *et al.*, 2010), with potential negative consequences in terms of their survival (Munday *et al.*, 2010). Recently, the kinematics and behaviour that underpin complex predator–prey interactions have also been used to characterize OA-related impairment (Allan *et al.*, 2013; Munday *et al.*, 2016; Allan *et al.*, 2017; McCormick *et al.*, 2018b). In the following section, we discuss the chronic and acute effects of OA on the kinematics and behaviour of fish escape responses, with considerations on predator–prey behaviours and their potential ecological consequences, while pointing out species-specific differences found in the responses. Research on the effect of OA on fish escape behaviour is relatively new compared to, for example, the effects of temperature. As a consequence, little is understood about the mechanisms underlying reported responses and the behavioural results are not always consistent among different species. This lack of consistency does not allow the development of hypothetical general scenarios of the effect of OA on predator–prey interactions, unlike the case of temperature and hypoxia.

### The effect of acidification on escape locomotion

**Figure 5:**
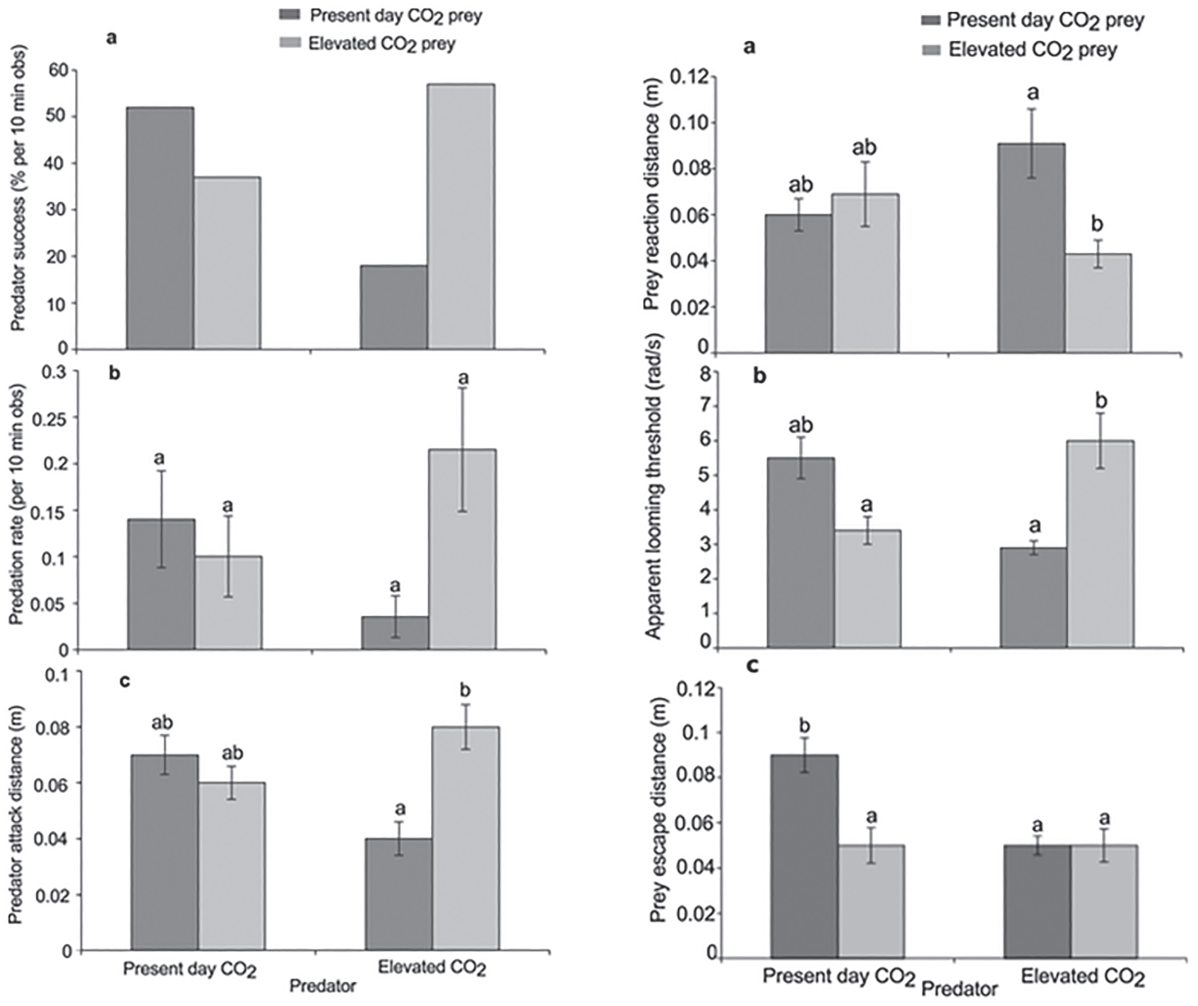
The effect of elevated CO_2_ on predator attack behaviour [left panel, (**a**) predator success, (**b**) predation rate, (**c**) predator attack distance] and prey escape response [right panel, (**a**) prey reaction distance, (**b**) apparent looming threshold, (**c**) prey escape distance]. Predators and prey were exposed to elevated CO_2_ (880 μatm) or a present-day control (440 μatm). The prey is the planktivorous damselfish *P. amboinensis* and the predator is the piscivorous dottyback *P. fuscus*, from Allan *et al.* (2013).

Similar to temperature and hypoxia effects, exposure to both chronic and acute high CO_2_ levels can affect locomotor performance, but whether the effect is negative or positive depends largely on the underlying physiology of the animal being tested. In addition, elevated CO_2_ levels can cause changes in aerobic scope (Couturier *et al.*, 2013; Rummer *et al.*, 2013) with direct implications on the functioning of predator–prey interactions (Rosa and Seibel, 2008; Couturier *et al.*, 2013). The way in which elevated CO_2_ affects aerobic scope has yielded contrasting results with some species showing an increase (Rummer *et al.*, 2013) and other species a decrease in aerobic scope (Munday *et al.*, 2009). The results of the effect of high CO_2_ on aerobic scope are potentially relevant for burst swimming, despite the fact that escape locomotion is powered anaerobically. This is because any effect of high CO_2_ conditions on aerobic scope may indirectly affect escape performance since the energy debt created by anaerobic activity has to be paid off by post-exercise oxygen consumption (Moyes *et al.*, 1993). However, in the Ambon damselfish (*P. amboinensis*), despite the finding of an increase in aerobic scope with increased CO_2_ (Couturier *et al.*, 2013), 4 days of exposure to high CO_2_ (~900 μatm) decreased escape locomotor performance (in terms of escape distance within a fixed time) (Allan *et al.*, 2013; Fig. 5). Similarly, a 4-day (~900 μatm) exposure to high CO_2_ negatively affected the escape swimming (mean and maximum speed) of the cinnamon clownfish (*Amphiprion melanopus*) (Allan *et al.*, 2014) and decreased the escape distance moved and average escape speed in the yellowtail kingfish (*Seriola lalandi*) [exposure period of 21 days post-hatch (~1000 μatm)] (Watson *et al.*, 2018). In contrast, Allan *et al.* (2017) found that there was no effect of high CO_2_ exposure (~900 μatm for 7 days) on escape distance, but there was a significant effect on escape speed in the Ward’s damselfish (*P. wardi*) (Allan *et al.*, 2017). There was also no effect of high CO_2_ exposure on the maximum bending angle and the duration of stage 1 in the escape response of marine medaka (*Oryzias melastigma*) following a 8–16-day embryonic exposure to two CO_2_ levels (~1100 and 1800 μatm) (Wang *et al.*, 2017). Therefore, although the escape swimming of some species appears to be affected by high CO_2_, the results are inconsistent among species, even when they belong to the same genus.

### The effects of acidification on non-locomotor variables of the escape response

The most significant impact of high CO_2_ exposure on escape responses appears to be related to the sensory performance that underpins successful escapes. If we consider the initial phase of a predator–prey interaction, which is often triggered by a visual cue, any process that alters this can alter the outcome of the interaction. High CO_2_ is known to affect the performance of various sensory channels including vision (Draper and Weissburg, 2019). For example, Chung and colleagues found that retinal flicker frequency was disrupted in the spiny damselfish (*Acanthochromis polyacanthus*) after a 6–7-day exposure to high CO_2_ (~900 μatm) (Chung *et al.*, 2014), which could have an impact on the ability of prey to visually detect approaching threats and the distance at which it responds to a high-speed predatory attack. Once an attack is perceived, the most important trait associated with a successful escape response is the reaction distance of the prey. Allan *et al.* (2013) showed that high CO_2_ exposure appears to reduce this distance as well as cause changes in ALT (*in sensu*Dill, 1974) in *P. amboinensis* attacked by real predators, leading to increased mortality (Fig. 5), although work based on predator–prey encounters using a different species from the same genus (*P. wardi*) showed no effect of high CO_2_ on either reaction distance or ALT (Allan *et al.*, 2017). Work using a mechanical stimulus showed that exposure to high CO_2_ negatively affected two fundamental traits of escape response, directionality and responsiveness, but had no effect on escape latency (Allan *et al.* 2014). Marine medaka (*Oryzias melastigma*) larvae showed no effect on escape latency but did exhibit a lower responsiveness to a mechano-sensory stimuli when reared in high CO_2_ compared to larvae reared under control conditions (Wang *et al.*, 2017). Wang and colleagues suggest that lower responsiveness in high CO_2_ may be due to an effect on the development of the ear (e.g. otoliths), the afferent connections in the VIIIth nerve and the inputs to the Mauthner cells.

The neural basis of high CO_2_ impairment of the escape response as found in some of the species investigated is unknown. It has been hypothesized that OA may induce changes in the sensory responsiveness of the Mauthner cell or other reticulospinal cells responsible for escapes (Allan *et al.*, 2013; Wang *et al.*, 2017). Previous work has demonstrated that the mechanism underlying behavioural malfunction in fishes exposed to elevated CO_2_ is due to disrupted neurotransmitter function, which is attributed to changes in the ion gradients over neuronal membranes (Nilsson *et al.*, 2012). This disruption has been suggested to lead to the excitation of GABA-A receptors (Nilsson *et al.*, 2012). Interestingly, Mauthner cells have GABA receptors distributed throughout them (Korn and Faber, 2005). GABA, acting primarily through interaction with GABA-A receptors, may cause Mauthner cell inhibition through membrane hyperpolarization and increased membrane conductance, which could potentially alter the response of the Mauthner cell (Lee *et al.*, 1993). Therefore, it is possible that the sensorimotor performance and the timing of the Mauthner cell’s firing in the more sensitive fish species are negatively affected by high-CO_2_ exposure, resulting in the misfiring and/or changes in the timing of the action potentials that generate escape response. This hypothesis is yet to be tested and would involve complex electrophysiology but presents an exciting avenue for future research. Allan and colleagues found that parental exposure to high CO_2_ (11 months at ~1000 μatm) reduced the negative effects of increased CO_2_ exposure on fish escape responses (i.e. both locomotor and non-locomotor traits), suggesting that trans-generational acclimation may occur. However, acclimation was not complete in escape speed and absent in directionality, suggesting that parental exposure does not completely compensate for the effects of high CO_2_ on escape performance (Allan *et al.*, 2014).

### Acidification effects on predator–prey encounters

To date, there are only a few studies that have examined the effects of CO_2_ exposure on both predator and prey attack and escape responses. Allan *et al.* (2013) found that although elevated CO_2_ had no effect on predator attack speed, the distance at which the predators started to strike at the prey was affected following high CO_2_ exposure (Fig. 5), suggesting an effect of high CO_2_ on motivation to feed. However, this effect differed depending on whether the prey had also been exposed to high CO_2_. A functional interpretation of the effects of CO_2_ exposure on predators is challenging because their response is a combination of their motivation to attack and the behaviour of the prey and because the neural mechanisms underpinning predatory attacks have not been studied as extensively as those for escape responses (Schriefer and Hale, 2004).

The dynamics and outcome of predator–prey encounters are likely to be dependent on the different sensitivity of individual species (predators and prey) to elevated CO_2_. For example, studies involving predators and prey exposed to increased CO_2_ have paired coral reef fish recruits with reef-based predators (Allan *et al.*, 2013). If we consider the differences between the pelagic environment inhabited by the recruits, and the reef environment, inhabited by the predators, these environments have very different levels of dissolved CO_2_. Coral reefs are dynamic shallow water habitats that experience diel cycles in CO_2_ due to the photosynthesis and respiration of corals. Therefore, it is possible that reef-based predators have already been exposed to high CO_2_ levels and may be displaying compensatory acclimation. This hypothesis is further supported by Jarrold and colleagues who showed that experimental exposure to diel CO_2_ cycles (42 dph at 1000 μatm ± 300 and 1000 ± 500 μatm) can reduce associated behavioural impairments in coral reef fish (Jarrold *et al.*, 2017). In addition to the effects on the kinematics underlying predator–prey interactions that occur at least in some species, one of the most consistent impairments due to increased CO_2_ levels is in the olfactory behaviour of species from multiple taxa (fish and invertebrates), across multiple ecosystems, responding maladaptively to predator odours and chemical alarm cues (for review, see Nagelkerken and Munday, 2016). A change in the olfactory landscape is a reliable indicator of a potential predatory attack. Therefore, misidentifying these important cues can lead to increased mortality rates (Ferrari *et al.*, 2011).

The results on the few predator–prey interactions presented here make it difficult to generalize across species, let alone across families; therefore, caution must be exercised when interpreting the ecological significance of these effects. Therefore, arguably, the effects of OA on fish escape responses and predator strikes, metabolism and hunger level are not sufficiently robust and the mechanistic basis not sufficiently understood, to be used for suggesting future scenarios at this time. More works on the mechanisms underpinning the effects of high CO_2_ and on a large number of species and species interactions are needed in order to increase our predictive power of the effect of high CO_2_ on predator–prey interactions. Increasing our understanding of the effects of high CO_2_ on predator–prey interactions is fundamental because these effects can cascade and result in changes in the species composition of communities, potentially disrupting the stability of the ecosystem (McCormick *et al.*, 2013; Nagelkerken *et al.*, 2018). Given that OA presents a significant and persistent environmental stressor, OA-induced maladaptive behaviours may cause future imbalances in predator–prey interactions. Therefore, knowing the physiological and neural basis of the effects of OA on predators and prey, and their relative adaptive potential, will allow us to better predict which species and which predator–prey interactions may be more sensitive than others.

## Multiple stressors

Single stressor experiments are typical of the early stages in the development of a research field, but these are soon followed by increasingly complex, multi-factorial experiments that attempt to incorporate additional layers of reality into the artificiality of manipulative experiments. Such experiments allow us to explore whether the stressors combine to yield additive, antagonistic or synergistic effects on the variables measured. To date, climate change experiments in the context of escape responses have been rare and involved binary combinations of stressors to mimic future environmental scenarios. The most commonly used combination of stressors has been water temperature and elevated CO_2_ levels. Previous meta-analyses have found that the interactions of elevated CO_2_ or hypoxia with elevated water temperature lead to synergistic effects on a range of physiological and life history characters (Harvey *et al.*, 2013; McBryan *et al.*, 2013), but studies have seldom examined the impacts of multiple stressors on escape responses. All meta-analyses conclude that we must be cautious in making inferences from single-stressor studies.

To date, only two studies have looked at the effect of elevated CO_2_ and temperature on the response of a fish to an artificial startle stimulus. Nasuchon and colleagues examined the escape response under elevated CO_2_ (400/1000 ppm) and temperature (15 and 19°C) of adult Japanese anchovy *Engraulis japonicus* acclimated for 1 month (Nasuchon *et al.*, 2016). Neither CO_2_ nor temperature affected the kinematic parameters analyzed (i.e. the escape trajectory, swimming velocity, acceleration, escape direction and frequency of bends), with the exception of the turning rate that was significantly higher at 19°C than at 15°C. Watson *et al.* (2018) used a fully factorial design (27 and 30°C and 405 and 930 ppm CO_2_) to test the combined effects of projected climate change on the escape response of larval kingfish (*Seriola lalandi*). Here, embryos and larvae were reared under the different environmental conditions until testing at 21 days post-hatch. High temperature improved performance by increasing responsiveness and maximum speed and decreasing escape latency. However, these effects were largely driven by a temperature-related increase in body mass and thus a more developmentally advanced stage for those individuals exposed to the higher temperature. On the other hand, high CO_2_ decreased distance covered and average speed during the escape but had no effect on responsiveness nor escape latency (Watson *et al*., 2018). There was no interaction between CO_2_ and temperature for any of the variables measured. These studies suggest there are likely to be species-specific differences in the response of fishes to the interacting effects of CO_2_ and temperature. Clearly, further examples are required before we can determine whether there is any phylogenetic pattern to the impact of these factors on escape performance.

**Figure 6:**
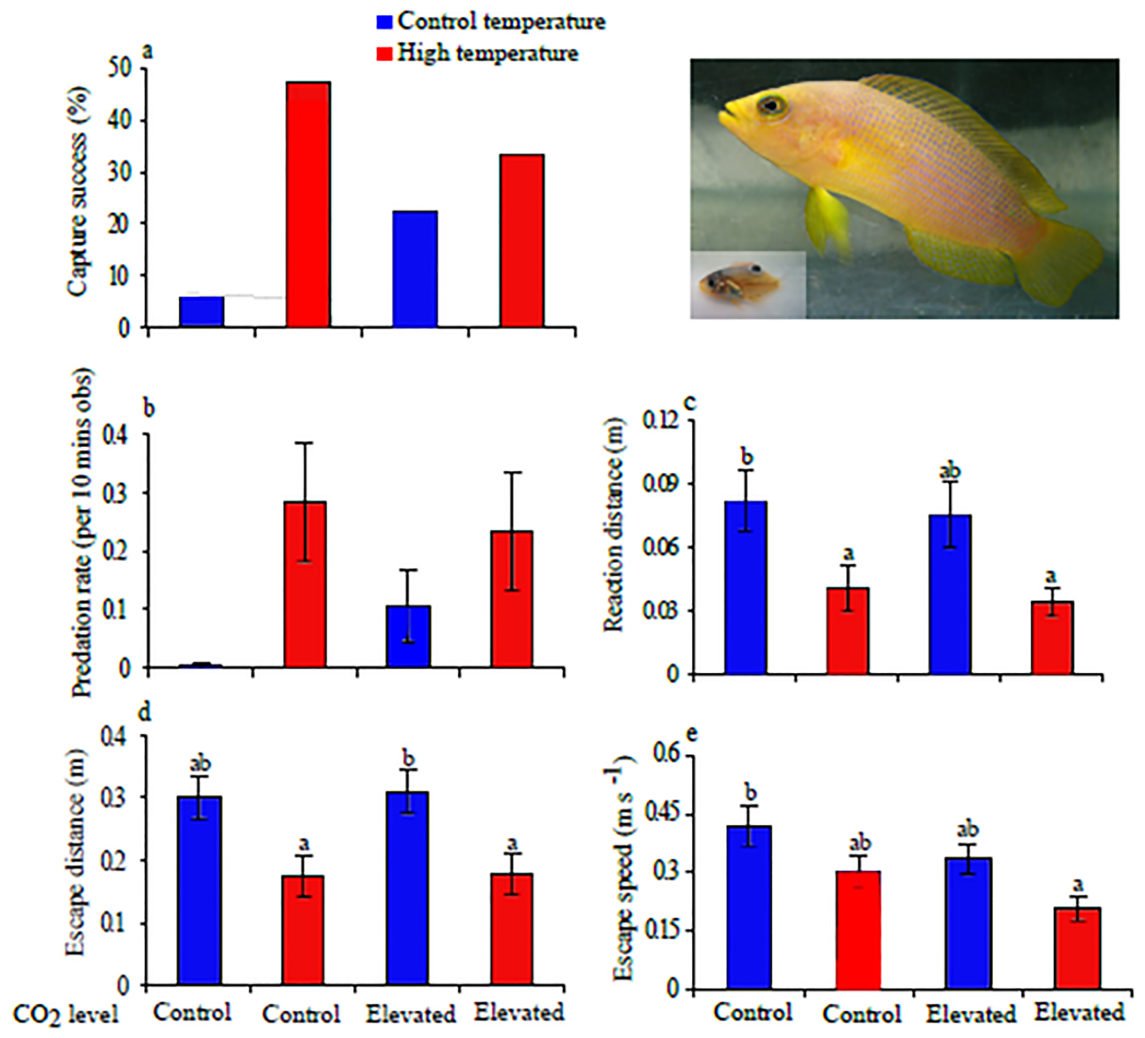
The effect of temperature (27 and 30°C) and elevated CO_2_ (405 and 930 μatm) on the interaction between dottyback predator (*P. fuscus*) and its damselfish prey (*P. wardi*); (**a**) capture success, (**b**) predation rate, (**c**) prey reaction distance, (**d**) escape distance and (**e**) escape speed [modified version from Allan *et al*. (2017)].

An important step towards understanding the impact of climate change at the community level is to explore predator–prey interactions from the perspective of both participants at the same time. Only one study has examined both the responses of the prey and the predator to combined future stressors. Allan and colleagues exposed newly settled damselfish (*P. wardi*) and predatory dottybacks (*P. fuscus*) to control or elevated CO_2_ (~405 and 930 μatm) and temperature (27 and 30°C) conditions for 7 days in a 2 × 2 design. Detailed examination of the interaction between predator and prey found that high temperature had an overwhelming effect on the escape behaviour of the prey (i.e. on reaction distance, ALT, escape distance, but not escape speed) compared with the combined exposure to elevated CO_2_ and high temperature (significant interaction effect on escape speed), or the independent effect of elevated CO_2_ (which affected only escape speed) (Fig. 6). Exposure to high temperatures led to increased capture success and predation rate. Interestingly, there was little influence of elevated CO_2_ on the behaviour of the predator, emphasizing the species specificity of the response to elevated CO_2_ and the pressing requirement to examine fish escape responses in an ecological context within future climate scenarios (Allan *et al.*, 2017).

Recently, studies have begun to incorporate natural cycles within climate variables, such as O_2_, CO_2_ or temperature. In the only study to date to attempt to investigate the effect of diel cycles of CO_2_ on fish anti-predator performance (Jarrold and Munday, 2018), the escape response to a startle stimulus of juvenile damselfish (*Acanthochromis polyacanthus*) was examined. Fish were reared for 11 weeks in two stable (450 and 1000 μatm) and two diel-cycling elevated CO_2_ treatments (1000 ± 300 and 1000 ± 500 μatm) at both current-day (29°C) and projected future temperatures (31°C). No interaction between CO_2_ and temperature for any performance characteristics was found, though survival was affected. Survival was lower in the two cycling CO_2_ treatments at 31°C compared with 29°C, but did not differ between temperatures in the two stable CO_2_ treatments. While temperature had significant independent effects on escape performance traits, there was no effect of stable or cycling CO_2_ levels nor was there an interaction between temperature and CO_2_.These findings support the idea that water temperature, confined within realistic near-future projections, has the greatest impact on escape performance compared to elevated CO_2_ (Allan *et al.*, 2017, Jarrold and Munday, 2018). Other stressors, such as dissolved oxygen can also display major diel fluctuations (e.g. Domenici *et al.*, 2007a) and these are yet to be incorporated into experiments. Although experiments that incorporate cycles in more than one stressor have yet to be undertaken, these will be important as diel temperature cycles peak during the daylight hours, while diel CO_2_ cycles peak at night, and oxygen levels tend to be lowest at dawn. These diel patterns may complicate predictions about how they interact to affect fish performance.

## Conclusions and suggestions for further research

To date, experiments have been overly simplistic, which hampers the predictions of future community dynamics. With few exceptions, levels of experimental treatments have been static rather than naturally dynamic. Experiments will benefit from incorporating the interactive effects of two or more stressors, each with a minimum of three levels. Ideally, choice of levels for each stressor will be informed by information on species’ stressor-specific reaction norms for performance as this will enhance interpretation. While studies are becoming increasingly complex, researchers are yet to incorporate parental effects into studies of the interactive effects of climate stressors on escape performance. The way parents respond to their environment can shape offspring phenotypes, and these may help or hinder escape performance. Trans-generational experiments examining various traits including growth and metabolism have shown how parental exposure to high CO_2_ (Miller *et al.*, 2012; Allan *et al.*, 2014; Welch *et al.*, 2014; McMahon *et al.*, 2018) and temperature (Salinas and Munch, 2012; Donelson *et al.*, 2014; Shama *et al.*, 2014; Bernal *et al.*, 2018) affect offspring performance in isolation, but it is not known how interactions between these stressors may modify parental effects and offspring responses. Clearly, experiments that span multiple generations and incorporate two or more stressors are needed to provide an understanding of the role of parental inputs on fishes under climate change. We need to understand the mechanisms whereby performance is actually being affected by the stressors and their interactions if we are to understand the species capacity to adapt or acclimate to these conditions.

Where possible, studies should ideally make a conscious and argued decision on the duration of exposure to the various stressors and findings and this should be put in relation to the magnitude of natural fluctuations in the environmental stressors within the study system, and their predicted changes in the future. For instance, temperatures change naturally in a hierarchy of temporal scales, with acute changes (maybe tidal or daily) within a more slowly changing average (seasonal) temperature signal. The capacity of an organism to cope with and respond to acute changes in environmental variables may then be just as important as their capacity to adjust through acclimation to seasonally driven cycles.

Empirical studies on various taxa that have been sampled across a species geographic range have shown that sensitivity to elevated CO_2_ conditions is linked to the local conditions experienced (Kelly *et al.*, 2013; Pespeni *et al.*, 2013; Thomsen *et al.*, 2017; Vargas *et al.*, 2017). Similarly, temperature and hypoxia tolerances are species and site specific (Bickler and Buck, 2007; Payne *et al.*, 2016). A meta-analysis of the impact of multiple stressors found that synergistic interactions may be quite common in nature, particularly where two or more stressors co-occur (Crain *et al.*, 2008). Their analysis found that when a third stressor was added to an experiment, it changed interaction effects in two-thirds of the experiments and doubled the number of synergistic interactions. It is likely that future meta-analyses of the influence of stressors on escape response and predator–prey interactions in general will find similar levels of complex interactive effects.

Modelling the effects of changes in temperature, oxygen and CO_2_ levels on predator–prey interactions is a challenging but a much needed task in order to increase our predictive ability of the ecological effects of climate change. The importance of these effects in shaping marine communities is highlighted by the differential outcomes that climate change may have on various species and taxa of predators and prey (Domenici *et al.*, 2007a), which can be modelled conceptually (Öhlund *et al.*, 2015) and metabolically (Dell *et al.*, 2014; Grady *et al.*, 2019). A promising avenue to gain predictive power on the ecological effects of climate change is that of focusing on the more obvious differences between various taxa of predators and prey. Endotherms and ectotherms will respond differently to climate change, and whether they are predators or prey will have consequences for the outcome of interactions that will affect the patterns of species abundance and community composition, with high relevance for conservation and mitigation strategies (Dell *et al.*, 2014; Grady *et al.*, 2019). Similarly, how body size may modulate the effect of climate change (e.g. small fish may be more hypoxia tolerant than large fish) (Pan *et al.*, 2016) is an interesting area for future research. Furthering our understanding of how climate change stressors affect escape responses in fish of different species and sizes is a fundamental step to scale up to predator–prey interactions and their consequences at the community level.
